# Establishing RNAi for basic research and pest control and identification of the most efficient target genes for pest control: a brief guide

**DOI:** 10.1186/s12983-021-00444-7

**Published:** 2021-12-04

**Authors:** Sonja Mehlhorn, Vera S. Hunnekuhl, Sven Geibel, Ralf Nauen, Gregor Bucher

**Affiliations:** 1 Crop Science Division, Bayer AG, R&D, Pest Control, Alfred-Nobel-Straße 50, 40789 Monheim, Germany; 2 Department of Evolutionary Developmental Genetics, Johann-Friedrich-Blumenbach Institute, GZMB, University of Göttingen, Göttingen, Germany

**Keywords:** RNA interference, RNAi, Establishment of RNAi, Off target control, Controls, Variability, Pest control

## Abstract

RNA interference (RNAi) has emerged as a powerful tool for knocking-down gene function in diverse taxa including arthropods for both basic biological research and application in pest control. The conservation of the RNAi mechanism in eukaryotes suggested that it should—in principle—be applicable to most arthropods. However, practical hurdles have been limiting the application in many taxa. For instance, species differ considerably with respect to efficiency of dsRNA uptake from the hemolymph or the gut. Here, we review some of the most frequently encountered technical obstacles when establishing RNAi and suggest a robust procedure for establishing this technique in insect species with special reference to pests. Finally, we present an approach to identify the most effective target genes for the potential control of agricultural and public health pests by RNAi.

## RNA interference in research and pest control

For a long time, studying gene function was restricted to highly developed model organisms such as *Drosophila melanogaster*, where genetic screens and the maintenance of mutants were eased by an elaborate genetic toolkit. The advent of reverse genetics based on RNA interference (RNAi) paved the way for selectively silencing genes outside established model organisms and has broadened the scope of gene function studies. The high conservation of this process throughout eukaryotes and the ease of application of double-stranded RNA (dsRNA) by injection without the need of elaborate genetic tools promise—in principle—to study gene function in any animal. However, significant hurdles have to be overcome to realize this potential and it has turned out that practical restrictions often hamper the establishment of the technique in organisms—both for basic research and pest control purposes. We review some of these hurdles and give hints that help establishing RNAi in a novel organism to foster the broad application of this technique.

RNAi is an ancestral immune response of eukaryotic organisms to combat viral infections, transposable elements and to regulate expression of endogenous genes [1–3]. It was first discovered in petunia plants where overexpression of an anthocyanin biosynthesis enzyme led to white or spotted flowers instead of the expected increase of color intensity [4]. Subsequently, spreading RNA was found to deplete homologous mRNA termed “posttranscriptional gene silencing” and showed its importance in virus resistance [4–8]. Similar observations in fungi were termed “quelling” [9, 10]. The identification of dsRNA as causative agent in *Caenorhabditis elegans* [11] and *Trypanosoma brucei* [12] led to the detailed molecular characterization of the RNAi mechanism, its ramifications and diversification between taxa [1–3, 13].

Here, we focus mainly on the application of RNAi in insects, where the gene inventory of RNAi components is highly conserved, indicating that—in theory—any insect should be amenable to this technique [13–15] but the same applies to other arthropods. Indeed, RNAi has been widely adopted to study gene function for purposes of basic research in arthropod species ranging from spiders and early branching hexapods to holometabolous insects [16–22] thereby opening new fields of research. For example, in the red flour beetle *Tribolium castaneum*, where RNAi is strong and easy to apply, a wide range of topics has been studied in terms of gene function ranging from embryonic and postembryonic development, evolution and physiology [16, 23–33].

Besides studying gene function in basic research, RNAi is being harnessed for eco-friendly and species-specific pest control [34–36]. Pre-and postharvest damage of agricultural crops by insect pests are thought to reduce yields by 18% despite preventive measures and economic losses are even expected to increase due to global warming [37–39]. So far, pest insect control has largely relied on chemical insecticides and BT toxin based approaches, yet the appearance of insecticide resistance and environmental and toxicological concerns called for the development of insecticides with novel modes of action and environmental benign profiles [38, 40–45]. RNAi has been successfully tested as means for controlling a number of pest species, particularly coleopteran pests [34–36, 46, 47].

Three main issues have emerged in endeavors to apply this technique: First, some insect clades appear to be less amenable to RNAi or RNAi via feeding than others and species-specific differences within clades are rather the rule than the exception. Second, efforts to establish RNAi have led to conflicting reports on whether RNAi works in a given species or not. It has remained unclear, in how far the divergent observations are due to biologically relevant strain-specific differences in RNAi susceptibility or whether technical issues have led to false positive reports. Third, dsRNA remains an expensive slowly acting chemical with often lower efficacy than classical pesticides. Hence, it is of crucial importance to identify the most efficient target genes and the best sequences targeting them. Based on success in one species, several RNAi target genes have been tested in different species for use in pest control. However, it has become clear recently that orthologous genes differ with respect to their efficacy between species [27, 48–55]. Alternative approaches are the prediction of essential genes supported by artificial intelligence [56] or large scale RNAi screens in model organisms and subsequent transfer to pest species [27, 32, 33].

Here, we first provide a brief overview on some of the reasons for differences of susceptibility to RNAi including biological variation and experimental issues. Based on this, we outline an approach to establish RNAi as a method in yet untested model species or insect pests. Then, we provide an efficient workflow for selecting most efficient RNAi target genes for pest control. Finally, we argue that optimizing the dsRNA fragment applied in pest control may further increase efficacy.

## Variability and experimental difficulties encountered with RNAi

### Delivery of dsRNA—environmental RNAi and putative dsRNA receptors

RNAi emerged early in evolution of eukaryotes as immune response against viruses and transposons and the core components of the RNAi machinery are widely conserved [1, 11–14, 57–60]. Therefore, it is expected that most if not all animal species will mount an RNAi response once dsRNA has reached the cytoplasm of their cells. However, the macromolecular dsRNA does not enter cells by itself calling for efficient delivery methods. Direct injection of dsRNA into cells is very efficient but feasible only during the first embryonic stages (often called embryonic RNAi) when the cells are still large. Application at later stages requires that dsRNA enters the cells after e.g. injection into the insect hemolymph. This can be advanced experimentally to some degree for instance by electroporation [61, 62] or by packaging the dsRNA into nanoparticles that facilitate entry into cells [48, 63–69].

A much more robust response is observed in species with an endogenous mechanism for dsRNA uptake, which is the basis for “environmental RNAi”, i.e. the uptake of dsRNA from the fluid surrounding cells [70]. If this feature is present, dsRNA can be injected into the hemolymph of any insect life stage and subsequent knock-down in most or all cells can be expected. Depending on the injected life stage, the application of dsRNA is often called “larval” or “pupal” or “adult RNAi” [71, 72]. Another option opened by environmental RNAi is “in vitro RNAi” knocking down gene function by soaking cells in culture [73]. In the nematode *C. elegans*, the molecular basis of environmental RNAi has been studied thoroughly [74–77] while in the fly *D. melanogaster*, environmental uptake of dsRNA appeared to be restricted to specific cell types and was studied in cell culture [78–81]. In other insect species, in contrast, environmental RNAi works efficiently [71, 82–85]. While the nature of the insect receptors remains disputed (see below) they appear to require dsRNAs with a minimum length of around 60 base pairs [86–89].

In the nematode *C. elegans* the membrane-spanning dsRNA-specific channel *systemic RNAi defective1* (SID1) was found to be essential for dsRNA uptake [74, 76, 90, 91]. However, no direct SID1 ortholog was found in insects [13] while the identified insect SID1-like (Sil) proteins showed more homology to the *C. elegans* Tag-130 / ChUP1 protein which, however, does not contribute to the RNAi response in *C. elegans* [13, 92]. Nevertheless, some Sil homologs were tested for their impact on systemic RNAi in a number of insect species—with varying results. In *Apis mellifera*, a Sil-protein was upregulated after dsRNA exposure [93] and in *Leptinotarsa decemlineata*, minor contribution to systemic RNAi were attributed to one of its Sil proteins (SilC but not SilA) [94, 95] while strong participation was found in *Diabrotica virgifera virgifera* [87, 96]. In *T. castaneum*, all three identified Sil proteins were irrelevant for systemic RNAi [13] and similar results were obtained for *Schistocerca gregaria* [92], *Plutella xylostella* [97] and *Locusta migratoria* [98]. Hence, Sid-1 like genes do not or at least not exclusively explain dsRNA uptake in insects.

Clathrin-dependent endocytosis is required for dsRNA uptake as well, for instance as part of the mechanism for SID2 mediated dsRNA entry from the gut lumen in *C. elegans* [99] and in a *D. melanogaster* cell line [80, 81]. This was confirmed for several insect species such as *L. decemlineata* [94], *Bactrocera dorsalis* [100], *T. castaneum* [101], *D. v. virgifera* [96] and *S. gregaria* [92]. Additional dsRNA receptors were proposed including pattern-recognition receptors [80] previously only associated with bacterial infections in *D. melanogaster*, and the scavenger receptors (SR) SR-CI and Eater [81, 102, 103]. Indeed, SRs were also found to be relevant for RNAi in *S. gregaria* and *L. decemlineata* [92, 95] and were upregulated after dsRNA exposure in honey bees [104, 105]. The fact that environmental RNAi does not appear to work in *D. melanogaster* [78] despite the presence of the endocytosis pathway indicates that another, yet unknown dsRNA receptor may exist in insects.

### Variability of RNAi: population specific differences or false positive reports?

For the application in pest control, environmental RNAi is a prerequisite because dsRNA needs to be taken up from the gut after oral uptake. However, trials with oral uptake of dsRNA often have limited success, and bioavailability of dsRNA remains the most critical issue for its application in pest control. Amenability to oral RNAi appears to be a species-specific property where species of some clades appear to be amenable to RNAi via feeding with higher likelihood than others. For example, a number of beetles exhibit a robust RNAi response after oral dsRNA uptake [27, 34, 51, 55, 106]. In contrast, many Lepidopterans were found to be quite resistant to environmental RNAi [86, 107]. There are positive reports for several stinkbugs [52, 108–110] while there are more mixed reports with respect to whiteflies [65, 111–114], aphids [115–120] and spider mites [121, 122]. Though RNAi was shown to work in several hemipteran pest species, its commercial use to target these species is currently out of scope due to the high concentrations of dsRNA necessary for a rather moderate response. Similarly, major constraints on the potential of RNAi to control mosquito vectors of human diseases such as anopheline species transmitting malaria were reported, particularly due to the need of rather high doses of dsRNA required for successful RNAi under applied conditions [123]. But even within a certain clade the efficiency can vary. For instance, embryonic injection of dsRNA leads to a quite robust response in *Bombyx mori* but fails to do so in *Spodoptera exigua* and hemolymph dsRNA injection led to effects in Saturniidae but not in Papilionoidae [107]. One exception within beetles are weevils, where several species such as the cotton boll weevil proved resilient to oral RNAi mediated control [124–127]. The picture is further complicated by contradicting reports on RNAi after oral uptake for closely related or even identical species. For instance, RNAi after oral feeding of dsRNA was reported for the red flour beetle *T. castaneum* in several studies [120, 128–130] while respective attempts in a number of other labs remained unsuccessful (G.B., Ernst A. Wimmer and Yoshinori Tomoyasu, personal information). Likewise, successful RNAi by feeding in aphids was reported [131, 132] while other work shows that dsRNA endonucleases in gut and hemolymph hamper efficient RNAi [117] and finally, RNAi by feeding was reported to work in honey bee larvae [133] while others found low efficiency when feeding larvae [134].

These observations could reflect either biological divergence among populations of one species or false positive data. Biological variability between strains of the same species would have important bearings on application. Most importantly, such genetic diversity would represent material from which resistance against dsRNA as pesticide could develop. Unfortunately, only few studies focus on this aspect. For instance, susceptibility to oral RNAi was compared among 14 European populations of the Colorado potato beetle revealing a degree of variability. However, this variability was comparably small and would probably not compromise RNAi efficacy under applied conditions—depending on recommended label rates [135]. Further, it was shown that RNAi resistance conferred by the inhibition of dsRNA uptake could be selected in field populations of the Western corn rootworm (Coleoptera) [136]. Unfortunately, this resistance mechanism most likely confers cross-resistance to any dsRNA, and would render the entire technology useless. A recent study in the pea aphid found high variability among strains and a subsequent genome wide association study revealed a number of unexpected candidate genes that might contribute to these differences [137]. In contrast, in the red flour beetle *T. castaneum*, similar strength of the RNAi response upon injection was found in several lab strains and in field strains [138, 139]. Taken together, some biological variability of dsRNA susceptibility can be expected between taxa and sometimes even among strains of one taxon but it does probably not explain the dramatic differences in reported RNAi efficacy.

False positive reports may be an alternative explanation for some diverging reports on RNAi efficiency. One reason is that that many studies on RNAi in pest control use lethality as readout for the RNAi treatment. However, this is a quite unspecific phenotype, which can occur due to many factors unrelated to RNAi treatment. For instance, problems with maintaining the insects, hidden infection with parasites, different age of the treated individuals, quality and purity of the injection needles, toxic substances remaining from dsRNA preparations etc., all increase the variability of the background lethality between experiments, therefore appropriate control treatments are of utmost importance. Indeed, sudden instances of increased lethality are observed even in well-established cultures of model insects from time to time. Pest species are often more difficult to maintain in the lab or even need to be collected from the field, adding an additional level of variability on any measure of survival. Even in the absence of a lethal effect of a given dsRNA treatment, this experimental variability combined with the unspecific nature of the readout will almost certainly lead to increased death in some experimental settings, which might be interpreted as positive results. Given their sometimes crucial relevance for publication and funding, the wish for positive results may sometimes be stronger than the tightness of the experimental controls. Therefore, we suggest using specific phenotypes to establish RNAi as a method and applying stringent controls that reveal confounding effects when studying lethality after RNAi (see below).

### Possible mechanisms leading to variability of the RNAi response

Research on the lack of RNAi after oral application has unveiled several possible modes of resistance. First, the midgut of lepidopteran insects, along with several orthopteran or hymenopteran species, is alkaline which destabilizes dsRNA and thus facilitates degradation [140–142]. Additionally, Lepidoptera-specific RNAi efficiency-related nucleases (REase) and other dsRNA nucleases expressed in the midgut degrade dsRNA before it can be taken up and processed by the RNAi machinery [143–147]. Quite often, dsRNA is eliminated in the hemolymph of Lepidoptera, Hemiptera and Orthoptera, or the saliva of Hemiptera. In many cases nucleases are suggested to be the primary cause of RNAi tolerance [52, 112, 117, 118, 130, 148–153]. In contrast, dsRNA was shown to be relatively stable in midgut and hemolymph of Dictyopterans, e.g. cockroaches [149, 154]. Finally, dsRNA was shown to be trapped in endosomes of *Heliothis virescens* and *Spodoptera frugiperda* cell lines and tissues, thus blocking further cleavage to small-interfering RNAs (siRNAs) [150, 155]. Several creative approaches have been developed to overcome dsRNA degradation in the gut [47]. For instance, liposomes have been used in Drosophilids and a stink bug [120, 156], cationic nanoparticles were used in *Ostrinia furnacalis* [157], chitosan nanoparticles enhanced the RNAi effect in the *Anopheles gambiae* and *Aedes aegypti* gut [69, 158], cell penetrating peptides fused to dsRNA binding domains were applied in *Anthonomus grandis* [159] while endosymbiont bacteria in the gut were used successfully to produce a stable dsRNA supply [160].

Viral infections are discussed as an additional mechanism leading to RNAi insensitivity. Some viruses have evolved protein effectors that suppress the RNAi response of their host. Persistent viral infection caused for example by the flock house virus, Drosophila C virus or Nora virus inhibited the RNAi machinery assembly or activity [161–164]. Moreover, the RNAi machinery could be overloaded or diverted by the expression of excess viral RNA thereby reducing the RNAi response to external dsRNAs [165, 166].

Another factor possibly influencing variability in oral dsRNA uptake could be the microbiome. In *P. versicolora*, RNAi efficiency was increased when bacteria inhabited their alimentary tract [54]. Especially *Pseudomonas putida* profited from the additional nutrition provided by dsRNA degradation products causing overgrowth and infection of the beetle [54]. The disruption of gut epithelia and translocation of bacteria accelerated the RNAi-induced mortality [54] and in *L. migratoria*, the gut microbiome was altered upon dsRNA injection [167].

## Establishing RNAi in a novel model organism or pest species

Establishing a robust procedure for the application of RNAi is of crucial importance for basic research in novel model organisms and for the development of RNAi-based pest control in pest species. In this section we present an approach that is based on our experience with these endeavors in beetles.

### The dsRNA fragment: length, selection, production, concentration

The length of the dsRNA fragment might be chosen between 500 and 1,000 bp. One reason is the cellular uptake mechanism, which in insects appears to require a certain minimal length of the dsRNA. For instance, 21mers or fragments of 31 bp length injected into the hemocoel did not elicit RNAi in the red flour beetle while fragments of 69 bp induced RNAi and dsRNA with the length of 520 bp was even more efficient [88]. In the Western corn root worm a dsRNA length of > 60 bp was required for an RNAi response after oral uptake [46, 89]. While dsRNA targeting *β-actin* of 60 bp length reduced target gene transcript levels in Colorado potato beetle, it was not sufficient to induce mortality which was only achieved by dsRNA fragments of > 200 bp length [168]. Whereas dsRNA uptake determines the minimum size there are also reasons for an upper limit. Fragments longer than 1.5 kb do sometimes anneal with low efficiency, such that the effective concentration of dsRNA may be low despite high concentration of RNA (G.B. unpublished observation). Besides, a length above 500 bp promises a more efficient in vitro dsRNA production (Ambion MEGAscript Kit manual).

In the cell, long dsRNA is processed into different siRNAs and depending on their sequence characteristics, some siRNAs have been shown to be more efficient than others. There are computational tools that predict the portion of efficient siRNAs and the portion of potential off target siRNAs and based on these data, the optimal fragment can be selected. The Deqor algorithm [169] was used in our large scale screen in *Tribolium* [32] and we indeed found that the optimized fragments in many cases resulted in stronger phenotypes compared to other fragments targeting the same gene (unquantified observation). An online version of the tool is found at https://www.eupheria.com/tools-resources/deqor/.

**Fig. 1:**
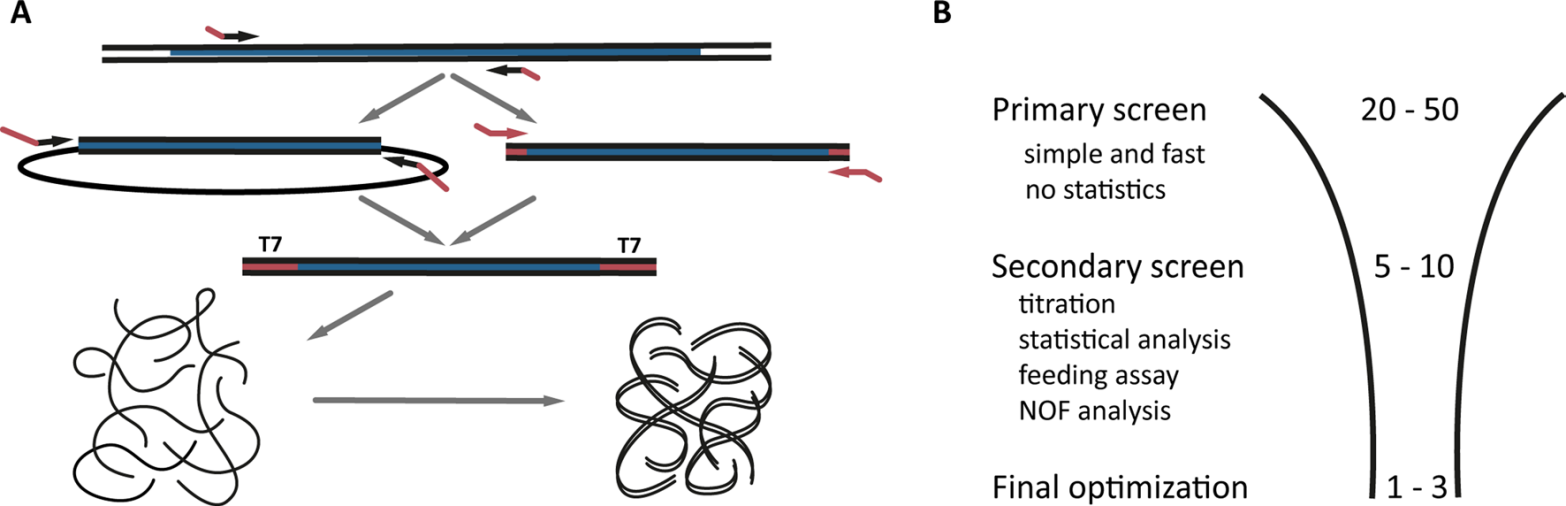
Workflows for dsRNA production and target gene screening. **a** A 500–1000 bp fragment of the target gene is amplified from genomic DNA or cDNA. Either, the fragment amplified with gene specific primers (black arrows) and cloned into a vector and confirmed by sequencing. Alternatively, linkers are attached to the primers (red part of the primers) and the PCR product is gel purified, sequenced and kept as stock. In both cases, primers that contain a T7 promoter sequence (red parts of arrows) are used to amplify the sequence in a secondary PCR. This PCR is used for in vitro transcription leading to a mixture of annealed and non-annealed RNA (left part). An annealing procedure enriches for double stranded RNAs. **b** Given the variability of orthologs to serve as good RNAi target genes for pest control, it is recommended to test a large number of genes in a primary screen, which is optimized for fast throughput. A small number of the best candidates is then further scrutinized by titration assays including statistical analysis, feeding assay and off target control using non-overlapping dsRNA fragments (NOF). Final optimization of the fragment might increase efficacy and safety in the field

In vitro dsRNA production is usually performed from a PCR template that has T7 promoter sequences at both ends. These T7 promoter sequences can be added by including them into the respective primers. However, in PCR reactions from complex DNA mixtures (like genomic DNA or cDNA), PCR artifact products are often produced along with the desired product. Such artifacts will be transcribed to dsRNA as well and will thereby reduce the effective amount of dsRNA and add off target effects to the specific effect of the target dsRNA. Therefore, a two-step process is advisable, where the target sequence is purified and sequenced (Fig. 1A). One option is to clone the fragment into a plasmid and confirm its correctness by sequencing before doing a secondary PCR with primers including T7 promoter sequences. Alternatively, the PCR product with linker sequences is excised from a gel, sequenced and stored (the linker used in the *iBeetle* screen was a partial T7 promoter: 5’-CTCACTATAGGGAGA-3’). In a secondary PCR, this template is amplified using primers that bind to the linker and contain T7 sequences [32]. This product is purified and used for an in vitro transcription (e.g. MEGAscript). Note that LiCl used for precipitating dsRNA is interfering with the Hedgehog pathway and can therefore be toxic and induce phenotypes. Therefore, the respective washing steps need to be performed very carefully—especially when lethality is the expected phenotype.

A portion of the RNA already anneals during the in vitro reaction but single stranded RNA will be present as well. In order to maximize the presence of double stranded RNA, a specific annealing reaction is recommended, the effect of which should be monitored on a gel (e.g. place reaction for 5 min in a 94 °C heating block, take out block and let cool at room temperature for 40 min). Due to secondary structures, RNA fragments behave differently from DNA on standard agarose gels. Nevertheless, differences before and after annealing are usually visible. Please contact the authors for a protocol.

Usually, the strength of the phenotype correlates with the amount of injected dsRNA. Therefore, the initial experiments should include maximum dsRNA concentrations (e.g. 2–3 µg/µl—higher dsRNA concentrations are often more difficult to obtain and dsRNA viscosity increases with concentration). Once a phenotype is observed, the concentration can be reduced empirically to reduce potential unspecific lethality or secondary effects. Note that standard procedures measuring DNA or RNA do not work well for determining effective dsRNA concentrations because most of these techniques detect single stranded RNA and/or nucleotides as well. Actually, nucleotides are not reliably removed by LiCl precipitation and residual nucleotides may indicate a higher concentration in measurements than actually present.

Commercially produced dsRNA is an alternative especially when many different genes are to be tested and/or molecular biology is not well established in the lab (e.g. Eupheria Biotech GmbH, Dresden, Germany—see competing interest section).

### *T*arget genes for establishing RNAi

Given the risk of false positive results when using lethality as a readout, it is advisable to establish and optimize the RNAi technique based on a gene with a specific phenotypic response. A convincing case for an RNAi response can be made by observing an expected specific visible but non-lethal phenotype after silencing a gene. Pigmentation genes are good candidates for that purpose. For instance, *laccase* is an enzyme involved in melanin-related cuticle tanning and its knock-down led to reduced pigmentation in a number of insects [24, 170–173]. Another option is the *ebony* gene, which upon knock-down led to darkened cuticle in *Tribolium* and other insects [87, 174, 175]. Another kind of specific phenotypes is expected after knocking down certain developmental regulatory genes. For instance, *Distal-less* is a homeobox gene required for the development of distal parts of appendages in many arthropods and upon downregulation the distal parts of appendages are defective [71, 176–178]. *Ultrabithorax* is a homeobox gene, the knock-down of which led to clearly visible abnormalities in the trunk region of insects and crustaceans [179–181]. Of note, the duplication of genes may lead to mutual compensation such that the knock-down of one paralog may not elicit a full phenotype or none at all. Hence, the presence of only one copy of the target gene should be confirmed before it is used for establishing RNAi.

### Delivering dsRNA

Most if not all animals mount an RNAi response once dsRNA has entered the cells. Hence, the limitation is actually getting the dsRNA into the cells. Unfortunately for application in pest control, RNAi after feeding is the method with least likelihood to work and usually the one with weakest phenotypic outcome. Therefore, we suggest establishing RNAi with more robust dsRNA delivery methods, first.

Direct injection of dsRNA into cells is restricted to the freshly laid eggs because they are still comparably large. Moreover, before cellularization, the dsRNA does not have to pass membranes to distribute. After injection of dsRNA into the one- or two-cell stage or into the syncytial phase of early insect development, it is very likely that dsRNA will elicit a response. The above-mentioned developmental genes are excellent test genes for embryonic phenotypes while pigmentation of the freshly eclosed larvae may not always be strong enough to be seen. The main hurdle in this procedure is that-depending on the species-it may be a real challenge to establish a protocol for collection and treatment of early embryos such that they survive injection. Actually, establishing embryonic injection is the crucial hurdle not only for RNAi targeting of early stages but also for other key applications in gene function studies such as transposon-mediated transgenesis or genome editing.

Many but not all arthropods show environmental RNAi, i.e. cells take up dsRNA from the surrounding medium. This uptake process is well studied in the nematode *C. elegans* [74–77, 91, 182] but it remains open in how far those processes are similar in insects. To test for environmental RNAi, dsRNA is injected into larvae or pupae and the phenotype is observed subsequently in the injected animal. Pigmentation phenotypes are an excellent choice for testing postembryonic stages but the phenotypes of the mentioned developmental genes in most cases affect morphology of pupae or adults as well. Due to the species-specific lag between time of injection and knock-down effect, and the difference in protein stability of different genes, it is advisable to perform time series injections in order to empirically determine the optimal timing for injection.

In some species, the knock-down effect is transmitted even to the offspring of injected females (parental RNAi) [71, 82, 83, 85, 183]. Once environmental RNAi has been shown to work by the above-mentioned experiment, this aspect can be tested by injection of female pupae or adults with dsRNA targeting developmental regulatory genes and scoring the hatchlings for morphological phenotypes. Note that hatchlings may not be pigmented enough to reliably score pigmentation genes and that injection of males has not led to transfer to the offspring so far [71].

### Quantification of the knock-down by qPCR or by the phenotypic readout?

qPCR is often used to confirm that the target gene is indeed downregulated. However, a number of issues limit the value of this control. First, there is no clearly defined relationship between the level of knock-down that has to be reached to elicit a phenotype—and the respective value varies from gene to gene. Actually, most genes are viable in a genetically heterozygous state, which means that 50% knock-down of transcripts is usually well tolerated or compensated for. As a rule of thumb, it is difficult to knock-down the transcript below 10–20% of residual mRNA while values above 30–40% residual mRNA may still lead to a strong phenotype, depending on the gene.

Second, many genes are part of regulatory feedback loops such that the transcript level will be determined by the primary downregulation by RNAi and modification by secondary effects. For instance, compensatory upregulation of paralogs or the targeted gene itself were observed after RNAi [138] (and unpublished observations V.H.). Compensatory upregulation of the transcript in the nucleus may increase the level of RNA detected by qPCR while downregulation in the cytoplasm may still be sufficient for a strong phenotype. For instance, we observed a very strong phenotype after RNAi targeting a developmental gene but surprisingly, qPCR indicated no strong modification of expression levels. However, qPCR measuring the intron of that gene indeed showed dramatic upregulation (unpublished observation). Likewise, we noted complex and unexpected patterns of down- and up-regulation with respect to several other genes after RNAi and these highly depended on the time between injection and qPCR.

Given these uncertainties regarding the interpretation of qPCR results, quantification of the phenotypic readout may still represent the most meaningful readout to answer the question of whether RNAi is an adequate tool for a specific gene in a given process.

### Sine qua non: off target controls

The result of any RNAi experiment can be blurred by off target effects. This means that the injected dsRNA does not exclusively target the intended transcript but may hit other transcripts as well. The presence of very short stretches of sequence identity (19 or more nucleotides) with an unrelated mRNA may already lead to efficient knock-down of that off-target mRNA [184]. The resulting phenotype then represents a combination of the intended effect and an off-target effect. Actually, in our large scale *iBeetle* screen (*T. castaneum*), we found qualitatively different phenotypes in 14% of all treatments even though bioinformatically optimized sequences were used [32]. Therefore, off target controls are absolutely essential in order to minimize the likelihood of erroneously assigning functions to certain genes. One measure is to identify sequences with identity to other mRNAs of 19 bp length by bioinformatics means and exclude them. Some online tools allow for checking long dsRNAs for off target effects (e.g. dsCheck, Deqor, SnapDragon, E-RNAi) but often the number of species is limited with E-RNAi having the highest number of species included [169, 185]. An essential control is in any case that the target mRNA is knocked down in an additional experiment with a second, non-overlapping fragment. Qualitatively coinciding phenotypes in both treatments indicate the absence of (at least strong) off target effects, while some degree of quantitative differences between fragments can occur.

## How to find the best RNAi target genes for pest control

### Varying efficacy of RNAi target genes across species

About 40% of all genes in an insect genome are essential, i.e. their knock-down leads to lethality of the animal at any stage [32, 186]. Hence, the problem is not to find an essential gene—the challenge is to identify one that leads to death most quickly at lowest doses of dsRNA. Some genes have been selected based on the assumption that they should be essential. Indeed, orthologs of some of these knowledge-based target genes have been used successfully tested in a number of different species (e.g. *V-ATPase, Act, tubulin*) [34, 49, 52, 113, 120]. However, our knowledge may not suffice to make reliable predictions on the best target genes for pest control. Efficacy is modulated by several unknown factors like stability of the protein (which is not affected by RNAi) and compensatory upregulation of the target gene or functionally related paralogs. Likewise, it is not clear, what cells should be targeted to induce most efficient death. Given these uncertainties, unbiased approaches can be used, where many genes are tested for their usability in pest control independent of assumptions. Due to the ease of rearing and application of RNAi, we have used the genetic model organism *T. castaneum* (red flour beetle) to score for lethality after RNAi of all genes in a completely unbiased approach [32, 33], providing a good base for the initial selection of target genes. Recent data showed that the efficacy of orthologous target genes can differ between taxa. For instance, RNAi in the red flour beetle silencing the previously used target genes *copi coatamere* and *V-ATPase* did induce lethality, but efficacy remained clearly below the level of the top 11 genes identified in that unbiased RNAi screen [33]. Meanwhile these top 11 genes have been tested in a number of other pest species to identify the most efficient target genes [27, 48–55]. The picture emerging from these studies (summarized in Table 1) is that usually at least one of the target genes efficient in one species turned out to be decent target genes in other pests as well.

**Table.1:** Comparison of studies using the eleven highly lethal genes found in *T. castaneum* by Ulrich et al. (2015). Key parameters of each study are indicated in the upper part of the table. Mortality rates [%] are shown for each of the eleven lethal genes, either estimated from figures (indicated with ~ before the number) or as stated in the publication. Mortalities of negative controls (GFP, YFP, IMPI) and positive controls (actin, IAP, V-ATPase) are included. Values of ≤ 40% are marked in red, ≥ 80% in blue to highlight inefficient and highly lethal genes, respectively

Species	*Phaedon cochleariae*		*Halyomorpha halys*		*Agrilus planipennis*		*Euschistus heros*	*Diabrotica virgifera*	*Brassicogethes aeneus*		*Dendroctonus frontalis*	*Anoplophora glabripennis*		*Plagiodera versicolora*	
Publication	Mehlhorn et al. (2021)		Mogilicherla et al. (2018)		Rodrigues et al. (2018)		Castellanos et al. (2019)	Knorr et al. (2018)	Knorr et al. (2018)		Kyre et al. (2019)	Dhandapani et al. (2020)		Zhang et al. (2019)	Xu et al. (2019)
Developmental stage	2nd instar larvae		Adults	2nd instar nymphs	Neonates	Adults	2nd instar nymphs	Neonates	Adults		Adults	4th/5th instar larvae	adults	1st instar	2nd instar larvae
Total no. of tested Genes	9	9	13	5	13	2	16	50	4	4	3	48	18	6	4
Delivery	Injection	Feeding	Injection	Feeding	Feeding	Feeding	Injection	Feeding	Injection	Feeding	Feeding	Injection	Injection	Feeding	Feeding
dsRNA amount	150 ng	0.3 µg/1 µg/ 3 µg per leaf disc	1 µg	20 µg-soaked bean pole, exchanged for 3d	10 µg/µl droplet for 4d	10 µg/µl	27.5 ng/mg body weight	500 ng/cm^2^ diet overlay	150 nl 250 ng/µl (=37.5ng)	Initial: 5 µl 1 µg/µl, then diet with 500 ng/cm2	10 µg	8 µg	10 µg	? Heat-inactivated bacteria producing dsRNA	8 ng/cm2, non-axenic
Time point	d10	d10	d7	d7	d8	d10	d7 / d14	d9	d14	d14	d10	d15	d15	d7	d7
*Cactus*	30-Jul	0/0/0	–	–	~43	–	~5/~67	–	–	–	–	0	–	~15	–
*srp54k*	100	100/100/100	~57	–	~48	–	~45/~98	–	–	–	–	0	–	75	100
*rop*	100	100/100/100	0	–	~40	–	–	82.8	~90	~90	–	0	–	–	–
*α-SNAP*	100	91.4/100/100	~43	–	~65	–	–	–	–	–	–	0	–	~60	100
*shibire*	63	11.4/0/0	~15	–	~80	~33	–	–	–	–	86.6	10	100	~10	?
*PP-α*	48	34.3/34.3 /45.7	100	~75	~67	–	~70/~96	–	–	–	–	0	–	–	–
*inr-a*	n.d.	n.d.	–	–	~72	–	~25/~30	51.89	–	–	–	0	–	–	–
*hsc70-3*	92.7	34.3/57.1/80	0	–	~92	~40	–	47.06	–	–	100	10	–	~38	–
*rpn7*	100	100/100/100	–	–	~62	–	~57/~95	59.2	–	–	–	0	–	–	–
*gawky*	n.d.	n.d.	–	–	~68	–	~50/~90	–	–	–	–	40	–	–	–
*rpt3*	100	100/100/100	–	–	~60	–	–	46.11	–	–	–	0	–	–	–
Negative control	3.7 (GFP)	*/ 2.3 (GFP)	*	*	~35 (GFP)	~10 (GFP)	~12/~18 (GFP)	11 (YFP)	~10 (IMPI)	~35 (IMPI)	~25	0 (GFP)	40 (GFP)	~15 (GFP)	*
*Actin*	–	–	~43	–	~55	–	~60/~98	–	–	–	–	50	40	82	100
*IAP*	–	–	~70	~75	~55	–	–	–	–	–	~30	100	100	–	–
*V-ATPase (various subunits)*	–	–	100	~10	–	–	~55/~85	–	–	–	–	50	20	–	–

*Control was used for mortality calculation; ? Gene was tested in the study, but data were not available yet; - gene was not tested in the study; n.d. not detected

### Challenges when testing RNAi by feeding

For application in pest control, dsRNA needs to be taken up by feeding. Hence, the target genes have ultimately to be tested in a feeding assay often using larvae as the most destructive life stage, at least for many chewing pests. Establishing a robust test is of key importance to avoid false positive results. First, the assay needs to ensure a baseline lethality of the treated insects, which is as low as possible and rather constant. Hence, an artificial diet must be developed that is based on well-defined components (for reproducibility), supports the regular development of the animals and does not interfere with dsRNA stability. For instance, culturing red flour beetle larvae in viscous flour-liquid mix can lead to increased death because these animals are adapted to dry habitats (unpublished observation). Herbivorous pest insects are best tested for RNAi efficacy using the respective host or test plants or parts thereof, e.g. by the spray application of dsRNA to leaf-discs as recently shown for mustard beetle larvae [51].

Second, degradation of dsRNA in the medium needs to be considered and ways to replenish the dsRNA have to be designed where necessary. Of note, dsRNA has been shown to be effective on leaves for up to several weeks under greenhouse conditions [46]. In our experiments using *Phaedon cochleariae* as a chrysomelid model beetle, we developed a spraying system that allowed administering defined amounts of dsRNA on leaves. Once the treated leaf-disc was consumed we supplied untreated leaf-discs [51].

### Design of the experiment and the controls

Orthologous target genes differ in their efficacy in different species (see above) and reliable predictors for the transferability of RNAi responses across species are still lacking. Therefore, it is advisable to start with a primary screen testing as many target genes as technically feasible (20 to 50 depending on the ease of application) at comparably high dsRNA concentrations. Based on the results of this primary screen, a smaller number of candidates (for instance the top 5 to 10) funnel into a secondary screen. Here, they are tested in extensive titration experiments where different concentrations of dsRNA are applied and the phenotypic effects are documented for an extended period of time. The most important phenotype is lethality, which is recorded over time to reveal the dynamics of the response. Additional parameters to document might include the number of molts, feeding behavior, weight, etc. The experiment could be complemented by qPCR analyses documenting the degree of knock-down. While the primary screen may still be based on a comparably low number of animals per treatment (depending on the baseline lethality e.g. 10 to 15), the subsequent experiments should be based on numbers that allow for statistical analyses, for instance at least three biological replicates with 9 or more animals per treatment. The concentrations should cover a broad range, which depends on the mode of application. For instance, we administered 3 µg, 1 µg and 0.3 µg per leaf-disc which was not completely consumed by several mustard beetle larvae feeding on it [51]. For the primary screen by injection in *T. castaneum*, we used five concentrations between 1 µg/µl to 3 ng/µl.

Given the likelihood of false positive results when using lethality as readout, a number of controls are essential. First, a dsRNA preparation of an non-endogenous gene (e.g. *EGFP*) should be used as negative control. This controls for unwanted effects by the dsRNA preparation and other technical factors (still, the dsRNA production of a target gene could be contaminated with LiCl or other toxic substances). Moreover, such a treatment controls for unspecific lethality induced by dsRNA (and its solvents) or by the artificial diet. Increased lethality can emerge from technical issues like toxic substances in the dsRNA preparation, infections of the animals or other problems with the culture (albeit less common). Hence, reproducing the result with an independent dsRNA preparation in an independent replicate is required to minimize false positive results. For the genes analyzed in detail, off-target effect with a non-overlapping fragment targeting the same gene need to be performed. Only genes that pass these controls should be considered for the laborious titrating experiment.

### Catalogue of potential target genes

The challenge of identifying the best RNAi target genes is restricted on the one hand by the limited level of transferability from one species to the other (see above). On the other hand, large scale screens are difficult to perform in most pest species. Hence, we suggest to perform a mini-screen with a selection of RNAi target genes identified in other studies [27, 32, 33, 187]. Depending on the experimental amenability of the species, a number of genes successfully shown to be lethal across a number of species (at least 10 but better 30–50 genes) should be tested to identify efficient target genes. A list of very good candidate genes based on a large scale RNAi screen in *T. castaneum* [33] and results of respective tests in other insects are given in Table 1. Meanwhile, we have completed this screen to cover the entire *T. castaneum* genome and the best target genes from this effort will be published in due time. Other genes frequently used targets are *V-ATPase* (although this turned out to be less efficient than the target genes identified in the screen) and *Actin,* while genes so far targeted in commercial products include *SSJ1* and *Snf7* [188, 189].

### Optimization of the target fragment for pest control

Besides the parameters for selection of a dsRNA fragment discussed above, there are additional considerations when it comes to the design of fragments for pest control. The fragment has to specifically target the pest but should not affect other species. Initial hopes to find species specific genes for pest control did not realize—because the most efficient target genes are highly conserved. Further, assuming 19 bp homology as the minimal sequence that would allow for knock-down, almost every long dsRNA sequence will have off target siRNAs that target other mRNAs. These off target sites are not restricted to the conserved regions of the protein [33]. Hence, for application in the field, the target mRNA should be searched for sequence stretches that do not show off-target sequences in non-target insects living in the same habitat. Alternatively, one might try to find stretches that target a number of related pest species while avoiding effects to non-target species. This could be especially relevant in integrated pest management programs, when dsRNA treatment is combined with the application of beneficial insectivorous species e.g. of the Coccinellidae (lady beetles) family. Tests on bee pollinators are mandatory for registration of insecticides [190] and are likely to become mandatory for insecticidal dsRNAs as well. Ecotoxicological effects on honey bees were evaluated as part of the risk assessment for Western corn rootworm *dssnf7* used in the first GM-crop with insecticidal dsRNA-traits approved by USA authorities [189, 191, 192]. However, accounts on RNAi efficiency e.g. in the honey bee *A. mellifera* are quite variable. It was shown that different dsRNA amounts were necessary to achieve knockdown of target genes with various effectiveness, an observation partially explained by differences in tissue susceptibility [93, 193, 194]. When choosing a worst case scenario with ds*vATPaseA* from Western corn rootworm with the highest homology to honey bee, no adverse effects were found [134]. In fact, even in case of full sequence identity with honey bee vATPaseA, survival of *A. mellifera* larvae and adults was not affected after dsRNA feeding [134].

In our hands, combining dsRNAs targeting different lethal genes lead to no synergistic effect. Our pairwise combinations of the top 11 target genes rather revealed additive effects [33]. However, co-administration of *hsc70-3* and *shi* dsRNA in *A. planipennis* larvae and adults suggested a synergistic effect on mortality [53], albeit at low levels.

Unfortunately, systematic work on designing and tinkering the optimal dsRNA sequence is still missing and potentially, ways of increasing efficacy remain to be discovered.

## Outlook: where might RNAi help in pest control?

The great promise of RNAi in pest control is the very species-specific action combined with the fact that dsRNA is a natural compound that quickly degrades in nature. So far, insect pest control by insecticidal dsRNAs is still on the verge of commercialization. To date, only one product, the transgenic GM-maize SmartStax PRO expressing double stranded RNA targeting *Dv-snf7* and *Bacillus thuringiensis* (Bt) insecticidal proteins (Head et al., 2017), gained approval and awaits its launch in the United States of America [191]. Another transgenic product based on RNAi mediated knock-down of the *Dv-SSJ1* gene has been tested [188, 195]. An alternative approach is the non-transgenic sprayable RNAi, which circumvents several problems of GM crops particularly in the EU such as lacking public support, long registration processes or difficulties in the efficient generation of transgenic plants [196]. Considering the fast and complete degradation of dsRNA in the environment suggest low environmental and health risks [197–199], this might be especially attractive for organic farming.

Unfortunately, there are also issues that hamper RNAi applications. First, RNAi as a control strategy, is comparably slow and taking several days, during which the insects can still damage the crop or transmit diseases. For this reason, dsRNA is less suitable for insect control in ornamental plant or cut flower cultures where pristine appearance is desired. Another drawback is the limited number of insect species that are amenable to RNAi by feeding. Formulations of dsRNA with nanoparticles aimed to improve oral delivery [48, 63, 64, 69, 157, 200]. While these formulations show some promise, they may not be available to organic farming and need to be tested for safety.

dsRNA products are still considered rather expensive compared to chemical insecticides. Advances in the production of long dsRNA considerably dropped production costs below 0.5$/g [201] and the results from [51] indicated that field rates of 10 g/ha may be enough leading to an estimation of production costs of at least 5$ per hectare (Note that this estimation does not cover development, distribution and profit). Additional costs will have to be covered by the price of the marketed product. This is high when for example compared to the chemical insecticide “Decis forte” formulation with the pyrethroid deltamethrin as active ingredient, which costs approximately 50–60 €/l depending on the vendor (e.g. [202, 203]). This corresponds to roughly 3–4 € per hectare at a field application rate of 5–7.5 g/ha [204–206]. On the other hand, the price of dsRNA insecticides will be competitive with other products like the Bt toxin-based “Xentari”, which costs about 20–40 € per hectare and needs application rates of at least 324 g per hectare on cabbage, root vegetable or tomato [207–211]. The issue of fast degradation of dsRNA may be less of an issue in greenhouse-grown crops because one predominant source of degradation—UV light—is strongly reduced. Hence, sprayable dsRNA products could be competitive in high value crop applications.

Taken together, the future of sprayable RNAi in agriculture is difficult to predict and the balance can still tip either way. It continues to face many limitations and therefore might end up as a niche product for specific pest control problems or as a resistance-management tool in agronomic settings with pest species, which developed high levels of resistance to conventional insecticides. Being a natural compound, the focus of insecticidal RNAi might well shift from agriculture to domestic insect nuisances such as ants and termites [212–214] and to mosquitoes as vectors of human diseases [215–217]. Hence, depending on the development of political and regulatory frameworks in Europe and other regions, RNAi may contribute to some agricultural and horticultural production systems and domestic uses as an alternative to chemical insecticides.

## Conclusions

RNAi has become a key technique for studying gene function outside the classic model organisms and it can be harnessed for species-specific and eco-friendly pest control. However, the optimal mode of dsRNA delivery, the strength of the gene knock-down and the degree of environmental uptake and systemic spread differ dramatically between taxa. Therefore, we present a guideline on how to establish the method in a careful and well-controlled way. In order to render RNAi mediated pest control economically interesting, it is essential to identify the most efficient target genes. We suggest that testing a moderate number of candidate target genes is required to find an optimal one.

## Data Availability

Not applicable.

## Abbreviations

RNAi: RNA interference
dsRNA: Double-stranded RNA
Sil: SID1-like gene
SR: Scavenger receptor
REase: RNAi efficiency-related nuclease
siRNA: Small-interfering RNA
Bt: *Bacillus thuringiensis*
